# Variations of rhizosphere and bulk soil microbial community in successive planting of Chinese fir (*Cunninghamia lanceolata*)

**DOI:** 10.3389/fpls.2022.954777

**Published:** 2022-08-12

**Authors:** Jiachen Chen, Zhifang Deng, Zheng Jiang, Jin Sun, Fangfang Meng, Xiaodong Zuo, Linkun Wu, Guangqiu Cao, Shijiang Cao

**Affiliations:** ^1^College of Forestry, Fujian Agriculture and Forestry University, Fuzhou, China; ^2^Chinese Fir Engineering Technology Research Center of the State Forestry and Grassland Administration, Fuzhou, China; ^3^Key Laboratory of Forest Stress Physiology, Ecology and Molecular Biology, Fuzhou, China; ^4^College of Life Sciences, Fujian Agriculture and Forestry University, Fuzhou, China; ^5^College of Agriculture, Fujian Agriculture and Forestry University, Fuzhou, China

**Keywords:** successive planting, soil microbial community, high-throughput sequencing, soil nutrient environment, Chinese fir

## Abstract

Successive planting and monoculture, as common forest management methods, are widely used globally, especially in Chinese fir plantations in the subtropical areas of southern China. Although soil fertility depletion and productivity decline caused by successive planting have been widely reported, the underlying mechanism is still ambiguous. In this study, the composition and diversity of soil microorganisms (rhizosphere and bulk soils) in Chinese fir seedlings exposed to successive planting soils (first-generation Chinese fir seedings, FCP. second-generation Chinese fir seedings, SCP. third-generation Chinese fir seedings, TCP) and broadleaf tree species soil (*Phoebe zhennan* S. Lee et F. N. Wei, CK) were examined with high-throughput sequencing technology. Our findings revealed that the diversity and richness of bacterial and fungal communities were remarkably reduced in TCP than FCP and SCP, and were remarkably different between FCP and SCP. At the phylum level, the fungi with greatest relative abundance were Basidiomycota (5.74–32.88%) and Ascomycota (57.63–87.38%), while the bacteria with the greatest relative abundance were Acidobacteria (23.16–31.17%) and Proteobacteria (24.71–29.32%) for all treatments in both soil types. Additionally, the relative abundance of some pathogens (*Penicillium* and *Burkholderia*) was significantly higher in TCP than in FCP and SCP, suggesting that the presence of pathogens is an important factor in increasing the incidence of soil-borne sickness. Moreover, changes in fungal and bacterial communities were predominantly driven by soil dissolved organic carbon (DOC), DOC/DON ratio (DOCN), NO_3_^–^-N, microbial biomass carbon (MBC), and MBC/MBN ratio (MBCN). Overall, the long-term monoculture of Chinese fir promotes the microecological imbalance of rhizosphere and bulk soil, and remarkably reduced soil microbial community diversity. These results can provide a scientific support for the implementation of future management measures for fir plantations (e.g., fertilization, addition of microbial fungicides, and construction of mixed forests).

## Introduction

Chinese fir (*Cunninghamia lanceolata* Lamb. Hook.) is considered one of the most valuable indigenous timber species in southern China owing to its stiff stem shape, outstanding timber characteristics, fast growth and high yield (Yan et al., 2021). The Ninth National Forest Resources Survey showed that the fir plantation forest has an area of 1.48 × 10^6^ ha and a storage volume of 7.75 × 10^9^ m^3^, covering for 1/4 and 1/3 of the national forest area and storage volume, respectively, both ranking first in China (Cui and Liu, 2020). The timber market is often short in supply with the rapid development of China’s economy. However, the main Chinese fir afforestation practice is to create single planting, successive planting, and short-term rotation with a limit of 20 or 25 years (Miao et al., 2019). These practices lead to many ecological problems, such as land failure and the decline of forest productivity (Tian et al., 2011a; Farooq et al., 2019). The causes for the reduction in productivity can be attributed to the loss of soil nutrition (Yang et al., 2016), the autotoxicity of root systems (Sun and He, 2019), and alterations in microbiological community structure (Wu et al., 2017). However, comprehensive and in-depth research on rhizosphere and bulk soil microbial communities and their interaction mechanisms in successively planted Chinese fir and its application to the management of fir plantations are lacking.

Many scholars worldwide have carried out much research on successive planting, but their results are not similar. For example, There was no decline in productivity between the second and third generations of *Pinus patula* plantations in the Swiss Usutu forest (Evans, 1999). The net primary productivity of Chinese fir has dropped remarkably between first rotation and second rotation (Tian et al., 2011b). A recent study showed that with the increase of stand age, the nitrogen cycling in *C. lanceolata* plantations is determined by biological processes (Ma et al., 2007; Wu et al., 2017).

Microbes are very sensitive to variations in the environment and available nutrients (Huhe et al., 2017). Xia et al. (2012) found that the content of MBC and the quantities of bacteria in bulk soil decreased significantly compared with rhizosphere soil in Chinese fir plantations. Meanwhile, the increase of pathogenic fungal caused by Chinese fir replant has also been reported. Luo et al. (2020) found that the number of *Fusarium* in rhizosphere soil of *Cunninghamia lanceolata* increased significantly with the increase of successive planting algebra. The successive planting of Chinese fir decreases soil pH, available phosphorus (AP), available nitrogen (AN), soil microbial biomass carbon (MBC), and microbial biomass nitrogen (MBN) (Xian et al., 2020), which may lead to changes in microbial community structure and diversity. Liu et al. (2010) showed that continuous planting of fir altered microbial communities related to nitrogen cycling and two functional genes (by DGGE), the Shannon index revealed that monospecific *C. lanceolata* plantations had lower bacterial diversity and two functional gene diversities (*nifH* and *amoA*) than mixed stands, but the opposite was observed for fungal diversity. Li et al. (2005) also found that bacterial and actinomycete populations decreased with successive plantings of fir, but fungal populations increased (DGGE). However, previous techniques for studying soil microbial composition in successive Chinese fir have focused on denaturing gradient gel electrophoresis (DGGE), phospholipid fatty acids (PLFAs), and community-level physiological profiles (Wu et al., 2017). Since the detection resolution of these technologies is severely constrained at the taxonomic level, it is not possible to perform extensive research on the soil microorganisms in the successive planting of Chinese fir.

Overall, climate (Yang and Wu, 2020), soil fertility (Camenzind et al., 2018), management measures (Hartmann et al., 2012), and geographical situation (Li et al., 2019) will affect the soil microbial community. Therefore, the results of successive planting on soil microbial community vary greatly because of the complexity and diversity of factors. For example, the successive planting of *Eucalyptus* reduced the number of soil microbial communities (measured by PLFA) and enzyme activity, but it eased with the increase in forest age (Chen et al., 2013). However, the diversity of soil fungi increased in *Casuarina equisetifolia* plantations (Zhou et al., 2019). In addition, autoinhibition and allelopathy caused by continuous planting of Chinese fir have also been reported. Chen et al. (2014) showed that the extracts of leaf litter, fine roots and root exudes had significant inhibition on the growth of Chinese fir germinant, and the inhibition increased with continuous planting. This inhibition effect was greater in rhizosphere soils than bulk soils.

This research applied a high-throughput sequencing method to explore the variations in rhizosphere and bulk soil microbial communities, clarify the characteristics of soil microbiome inside successive planting of Chinese fir, and reveal the main environmental factors that cause alterations in soil microbial communities. In particular, we supposed that: (1) successive planting will reduce the diversity of rhizosphere soil bacteria and fungi, and soil microbial community structure will also change accordingly; (2) changes in soil microbial community structure are related to soil nutrients [e.g., dissolved organic carbon (DOC), dissolved organic nitrogen (DON), available phosphorus (AP)] and/or environmental factors (e.g., bulk density, soil moisture content). Our study will advance knowledge of microbial diversity and community structure in response to successive planting and offer theoretical guidance for long-term management of Chinese fir plantations.

## Materials and methods

### Sampling site

**FIGURE 1:**
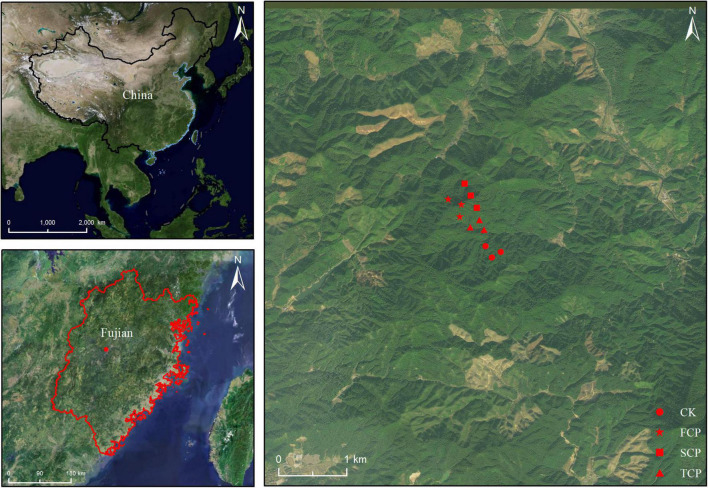
Location of soil sampling site with varying generations at the Wangtai Town, Nanping City, Fujian Province, China. CK, Nanmu plantation; FCP, first-generation plantation; SCP, second-generation plantation; TCP, third-generation plantation.

The soil sampling site was located in fir plantations with different successive generations in An Caoxia, Xiahou Village, Wangtai Town, Nanping City, Fujian Province (117° 57′ E, 26° 28′ N, Figure 1). The area has an altitude of about 200 m, a middle subtropical monsoon climate with a mean annual temperature of 18.9°C, total annual average precipitation of 1,969 mm, an average annual evaporation of 1,143 mm, and an average annual relative humidity of 83%. The soil is red soil developed by granite with a thickness above 100 cm and a loose surface layer. The first-generation fir plantation was planted in 1919 from clear cutting. The second-generation fir plantation, 500 m away from the first-generation fir plantation, was established in 1997 after slash burning first-generation fir plantation that was established in 1970 after the felling of evergreen broadleaved forests. The third-generation fir plantation, which is 1.3 km away from the first-generation fir plantation and 600 m away from the second-generation fir plantation, was established in 1996 after clear cutting the second-generation fir plantation that was built in 1971 after cutting the first-generation fir plantation, this plantation established from the natural evergreen broad-leaved forest in the 1930s. The *Phoebe zhennan* plantation was established in 1975 and is 1.2 km away from the first-generation fir plantation. Three sample plots (20 m × 20 m) were set up for each stand type to investigate stand basic information (Supplementary Table 1). Five sample points were selected for each sample plot by S-shaped sampling method. The top layer of dead branches and leaves were removed, and the soil in the 0–40 cm layer was collected and transported to Fujian Agriculture and Forestry University to be mixed and used as the substrate for potting experiments.

### Experimental design

The well-mixed successive planted fir soil was used as the culture substrate and was packed into pots of uniform size (28 cm × 30 cm). Fir seedlings of the same size (35–40 cm in height and 1.5–2.0 cm in diameter) were planted in pots. These fir seedlings are all excellent species selected by the subject group in the previous stage (Luo et al., 2019) and three replicates per treatment. Each replicate has 10 plants, total 120 plants. The cultivation site was the backyard of Laboratory 2, Science and Technology Park, Fujian Agriculture and Forestry University, and the cultivation period was 2 years, during which only normal watering and weeding management were carried out.

### Soil sample collection and labeling

Soil samples were taken after 2 years, the soils that are firmly attached to the root surface and collected after the roots have been shaken were identified as rhizosphere soils (Phillips and Fahey, 2006), while bulk soil was defined as root-free soil. Rhizosphere and bulk soil samples were labeled as “r” and “nr.”

### Soil sample analysis

#### Method for determining soil chemical properties

The collected soil samples were divided into three parts and exposed to 4°C (for soil DOC, DON, microbial biomass, NO_3_^–^-N), normal temperature (for soil TC, TN, AP, NH4^+^-N) and –80°C (for high-throughput sequencing). Soil total carbon (TC) and total nitrogen (TN) were examined using an element analyzer (VARIO MAX, Germany). Chloroform fumigation leaching was used to recover soil MBC and MBN (Vance et al., 1987). Soil mineral nitrogen (NH4^+^-N and NO_3_^––^N) was isolated by leaching with 1 M KCl solution and then determined by a fully automated interrupted chemical analyzer (Smartchem 200, AMSAliance, Italy). DOC and dissolved organic nitrogen (DON) were obtained by water leaching (2:1 water-to-soil ratio) and examined by a TOC analyzer equipped with a TN unit (TOC-LCPH, Shimadzu Scientific Instruments, Japan). Soil available phosphorus (AP) was determined by sodium hydroxide fusion-molybdenum antimony colorimetric (Liu et al., 2017).

#### Soil total DNA extraction

Using the PowerSoil^®^ DNA Isolation Kit from MOBIO, United States, the total DNA of soil microorganisms was extracted according to the manufacturer’s instructions. DNA quality was determined by combining NanoDrop (DNA purity assay), Qubit (DNA concentration assay), and agarose gel electrophoresis (RNA integrity assay).

#### PCR amplification of soil bacterial 16S rRNA and fungal ITS regions

The V3–V4 region of 16S rRNA was amplified using a specific primer with a barcode. The bacterial V3–V4 region primers were: 341F: CCTAYGGGRBGCASCAG and 806R: GGACTACNNGGGTATCTAAT. The fungal ITS2 region was amplified by using the specific primers: F: GCATCGATGAAGAACGCAGC and R: ATATGTAGGATGAAGAACGYAGYRAA.

Soil bacterial 16S rRNA (V3–V4) and the conserved fragments of the fungal ITS2 region were amplified. The extracted microbial genomic DNA was assayed for DNA concentration and quality by NanoDrop. The target fragments were amplified by a two-step PCR method. The first step of PCR amplification obtains the target fragment of the corresponding region. The PCR reaction system (25 μL) comprised 0.75 μL of forward primer, 0.75 μL of reverse primer, 0.5 μL of dNTP, 5 μL of GC, 5 μL of buffer, 0.1 μL of Q5 polymerase (NEB), and 12.9 μL of ddH_2_O. The PCR reaction procedure was: 95°C for 5 min; 15 cycles of 95°C for 1 min, 50°C for 1 min, and 72°C for 1 min; and an extension of 72°C for 7 min. The second step of PCR amplification adds the index to distinguish the samples. The PCR reaction program was: 10 cycles of 98°C for 30 s, 98°C for 10 s, 65°C for 30 s, and 72°C for 30 s and an extension of 72°C for 5 min. The amplified product was detected by 2% agarose gel electrophoresis. The PCR product was purified by AMpure magnetic beads, and NanoDrop was used for quantification and quality assessment. The samples were mixed with the same mass. Then, the target band was recovered by gelatinization. The mixed sample library was subjected to Agilent 2100 Bioanalyzer detection and quantitative PCR test and then sequenced using Hiseq2500 PE250.

### Data processing

UCLUST (Caporaso et al., 2010) in QIIME software (Edgar, 2010) was applied to aggregate the tags at 97% similarity level obtain operational taxonomic units (OTUs). Then, the OTUs were classified through the Silva taxonomic database to obtain species at each level composition and their relative abundance.

Alpha diversity (Shannon, Chao1, and ACE) was calculated by Mothur (V.1.34.0)^1^ (Kemp and Aller, 2004). The number of sequences present in the samples was clustered at the 97% similarity level to generate the community richness index (Chao1, ACE) and diversity index (Shannon), which were used to compare the diversity index between samples.

### Statistical analysis

Two-way analyses of variance (ANOVAs) were performed to determine the effects of successive generations, soil types (rhizosphere and bulk soil) and their interactions on soil abiotic properties and microbial attributes (OTUs, Shannon, ACE, Chao1). To determine the differences between soil abiotic characteristics, microbial biomass, microbial diversities, and relative abundance of microbial most abundant phyla, genus, and alpha diversity index, one-way analysis of variance (ANOVA) and Tukey’s honestly significant difference tests were performed. Using the “ape” and “vegan” packages in R software, principal coordinate analysis (PCoA) based on the Bray-Curtis distance, and PERMANOVA (Adonis) were used to visualize the community structure among different successive generations. Redundancy analysis (RDA) was utilized to elucidate the correlation between soil microbial composition with environmental factors based on OTU relative abundance data (Canoco5).^2^ Then, the significance of RDA correlations was examined using the Monte Carlo permutations test (999 permutations). All statistical analysis was completed by R v 3.6.2.

## Results

### Soil abiotic properties

Two-way ANOVA with repeated measurements showed a substantial connection among successive planting generations and soil types on soil nutrients, such as MBC and NO_3_^–^-N (Supplementary Table 2, *P* < 0.01). Different control practices (CK and replant) prominently changed the soil nutrients, except for TN, DON, and MBN. Soil types also affected DOC, DOCN, MBC, MBCN, NH4^+^-N, and AP (Supplementary Table 2, *P* < 0.01). In the rhizosphere soil, the TCN, MBCN, TC, TN, MBC, MBN, DON, and NO_3_^–^-N contents showed varying degrees of decline in TCP compared with FCP and SCP (Table 1). DON and NO_3_^–^-N contents of the TCP were significantly lower than in FCP by 49.18 and 53.62%. TC and TCN of the TCP were significantly lower than in the SCP by 41.30 and 51.13% (Table 1). In the bulk soil, although the TC, TCN, and DON decreased in varying degrees in TCP than in FCP, the differences were not noticeable. However, TC and TCN in TCP were remarkably lower than those in SCP, by 27.84 and 25.87%, respectively (Table 1). MBC, MBCN, and NO_3_^–^-N in SCP were notably lower than those in FCP (Table 1), by 106.31, 121.49, and 121.57%, respectively.

**TABLE 1:** Soil abiotic properties in Chinese fir seedings with different successive planting generations (rhizosphere and bulk soil).

Soil type	TC g/kg	TN g/kg	TCN	MBC mg/kg	MBN mg/kg	MBCN
FCP-r	35.97 ± 4.47ab	1.07 ± 0.18a	34.51 ± 8.67ab	608.94 ± 64.15a	123.36 ± 2.80a	4.95 ± 0.64a
SCP-r	41.23 ± 1.66a	0.98 ± 0.01a	42.15 ± 1.28a	429.9 ± 20.66b	127.90 ± 6.65a	3.37 ± 0.35b
TCP-r	29.18 ± 1.52b	1.05 ± 0.02a	27.89 ± 1.02b	520.87 ± 51.93ab	121.95 ± 4.29a	4.28 ± 0.57ab
CK-r	34.76 ± 2.30ab	1.06 ± 0.02a	32.87 ± 2.34b	642.50 ± 88.21a	129.55 ± 9.53a	5.00 ± 0.88a
FCP-nr	33.47 ± 2.14b	1.01 ± 0.02a	32.98 ± 1.97ab	311.90 ± 13.62a	117.02 ± 9.37a	2.68 ± 0.30a
SCP-nr	40.36 ± 1.89a	1.07 ± 0.02ab	37.80 ± 2.32a	151.18 ± 10.61b	125.53 ± 13.42a	1.21 ± 0.14b
TCP-nr	31.57 ± 3.14b	1.05 ± 0.01ab	30.03 ± 3.15b	380.70 ± 26.20a	123.08 ± 14.00a	3.12 ± 0.46a
CK-nr	33.10 ± 2.43b	1.06 ± 0.04b	31.40 ± 3.46b	371.29 ± 84.68a	132.71 ± 7.71a	2.80 ± 0.60a

**Soil type**	**DOC mg/kg**	**DON mg/kg**	**DOCN**	**NH_4_^+^-N mg/kg**	**NO_3_^–^-N mg/kg**	**AP mg/kg**

FCP-r	167.25 ± 5.10c	20.96 ± 1.06a	7.98 ± 0.17b	13.32 ± 0.78b	1.06 ± 0.06a	2.11 ± 0.27c
SCP-r	176.15 ± 9.52c	19.12 ± 1.51ab	9.23 ± 0.65b	18.56 ± 0.56a	0.41 ± 0.07c	2.18 ± 0.05c
TCP-r	202.43 ± 17.34b	14.05 ± 2.79c	14.75 ± 2.84a	18.09 ± 0.69a	0.69 ± 0.10b	4.47 ± 0.53b
CK-r	290.08 ± 2.75a	17.54 ± 0.56b	16.54 ± 0.56a	14.46 ± 0.38b	0.43 ± 0.11c	6.90 ± 0.16a
FCP-nr	208.52 ± 5.17b	17.94 ± 2.75ab	11.83 ± 2.04b	11.13 ± 0.026b	1.13 ± 0.05ab	2.54 ± 0.07c
SCP-nr	229.84 ± 7.83ab	11.71 ± 1.03b	19.68 ± 1.30a	16.85 ± 0.36a	0.51 ± 0.10c	2.47 ± 0.04c
TCP-nr	246.53 ± 8.78a	13.59 ± 2.57a	18.62 ± 3.88a	16.56 ± 0.16a	0.92 ± 0.15a	5.17 ± 0.16b
CK-nr	248.8 ± 3.91a	18.11 ± 3.15a	14.25 ± 4.51ab	11.17 ± 0.09b	0.76 ± 0.15b	7.26 ± 1.65a

TC, total carbon; TN, total nitrogen; TCN, TC/TN ratio; DOC, dissolved organic carbon; DON, dissolved organic nitrogen; DOCN, DOC/DON ratio; MBC, microbial biomass carbon; MBN, microbial biomass nitrogen; MBCN, MBC/MBN ratio; AP, available phosphorus; NH_4_^+^-N, ammonium nitrogen; NO_3_^–^-N, nitrate nitrogen CK, Nanmu seedings; FCP, first-generation seedings; SCP, second-generation seedings; TCP, third-generation seedings. r, rhizosphere soil; nr, bulk soil. Values are the means ± standard errors (n = 3). Different lowercase letters show significant differences (P < 0.05).

### Diversity of soil microbial community

A total of 338,331,182 and 29,688,104 high-quality 16S rRNA and ITS gene sequences were obtained by flowing quality filtering and chimera detection, and the average lengths of the sequences were 448 and 347, respectively. In all sample, the number of 16S rRNAs were 58,691–71,937, and the number of ITS varied from 12,976 to 17,449 (Supplementary Table 3). These sequences were categorized as OTUs (97% similarity). The OTU numbers of all soil bacterial and fungal samples were 5,408–9,631 and 970–1,533, respectively (Supplementary Table 3).

Two-way ANOVA with repeated measurements showed that successive planting generation and soil type affected the diversity indices of bacteria and fungi, such as Chao1, ACE, and Shannon indices (Supplementary Table 4). In the rhizosphere soil, except for the ACE index, Chao1 in TCP decreased significantly by 38.86% compared with FCP, whereas the Shannon index was markedly lesser in TCP compared with FCP and SCP, decreased by 1.61 and 5.01% (Table 2). The Shannon index of fungi was markedly lesser in TCP compared with FCP and SCP, decreased by 2.91 and 8.92%. Chao1 index, and ACE index were lower in TCP than in SCP, but no obviously change was observed between SCP and TCP apart from the OTUs (Table 3). In bulk soil, except for the Shannon index in TCP, which was considerably lower than that in SCP, the other indices did not substantially decrease in TCP than in SCP, but were lower in SCP than in FCP (Table 2). The fungal alpha diversity showed that the Shannon index, and ACE index in TCP were significantly lower than those in SCP (Table 3).

**TABLE 2:** Alpha diversity indices of soil bacteria in different successive planting generations.

Soil type	Shannon	Chao1	ACE
FCP-r	6.92 ± 0.07a	20,098 ± 4455a	29,654 ± 7801a
SCP-r	7.15 ± 0.01b	13,676 ± 998b	16,717 ± 1413a
TCP-r	6.81 ± 0.02c	14,474 ± 1483b	19,317 ± 2128a
CK-r	6.60 ± 001d	9,704 ± 700c	12,217 ± 884a
FCP-nr	7.02 ± 0.02a	14,573 ± 991ab	19,041 ± 782a
SCP-nr	7.12 ± 0.01b	13,392 ± 151a	16,797 ± 78a
TCP-nr	7.05 ± 0.01a	17,201 ± 465b	23,901 ± 1120a
CK-nr	6.70 ± 0.01c	13,611 ± 400a	18,047 ± 771a

OTUs, operation taxonomy units (97% similarity). Values are the means ± standard errors (n = 3). Different lowercase letters show significant differences (P < 0.05). CK, Nanmu seedings; FCP, first-generation seedings; SCP, second-generation seedings; TCP, third-generation seedings. r, rhizosphere soil; nr, bulk soil.

**TABLE 3:** Alpha diversity indices of soil fungi in different successive planting generations.

Soil type	Shannon	Chao1	ACE
FCP-r	4.96 ± 0.43b	2,035 ± 86.16b	3,007 ± 114.04b
SCP-r	5.25 ± 0.26a	3,085 ± 312.28a	4,948 ± 420.67a
TCP-r	4.82 ± 0.01c	2,783 ± 430.26a	4,145 ± 700.64a
CK-r	4.93 ± 0.60b	2,125 ± 254.72b	3,200 ± 302.76b
FCP-nr	4.38 ± 0.21c	2,382 ± 408.31b	4,036 ± 645.33b
SCP-nr	5.33 ± 0.78a	2,922 ± 196.90a	5,328 ± 687.33a
TCP-nr	4.60 ± 0.24b	2,535 ± 138.38ab	3,447 ± 136.89b
CK-nr	5.34 ± 0.29a	2,537 ± 132.28ab	3,585 ± 320.42b

OTUs, operation taxonomy units (97% similarity). Values are the means ± standard errors (n = 3). Different lowercase letters show significant differences (P < 0.05). CK, Nanmu seedings; FCP, first-generation seedings; SCP, second-generation seedings; TCP, third-generation seedings. r, rhizosphere soil; nr, bulk soil.

### Soil microbial community composition

**FIGURE 2:**
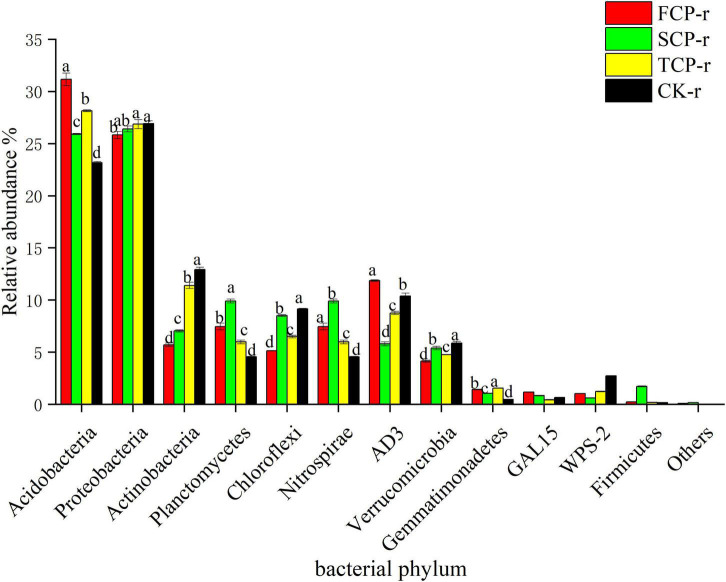
Relative abundance of predominant bacterial community in successive generations within rhizosphere soil at the phyla level. Other stands for undefined categories. Value show means ± standard error (*n* = 3). Different lowercase letters show significant differences. CK, Nanmu seedings; FCP, first-generation seedings; SCP, second-generation seedings; TCP, third-generation seedings. r, rhizosphere soil.

The bacterial communities with supreme relative abundances at the phylum level were Acidobacteria (23.17–31.17%) and Proteobacteria (24.71–29.31%), followed by Actinobacteria (5.72–14.85%) and Planctomycetes (4.55–12.51%) (Figure 2 and Supplementary Figure 1). The relative abundance of Acidobacteria was in the order: FCP > TCP > SCP > CK, and the difference between successive generations was significant (Figure 2). In comparison, the relative abundances of Proteobacteria and Actinobacteria showed an increasing trend with successive generations (Figure 2 and Supplementary Figure 1).

**FIGURE 3:**
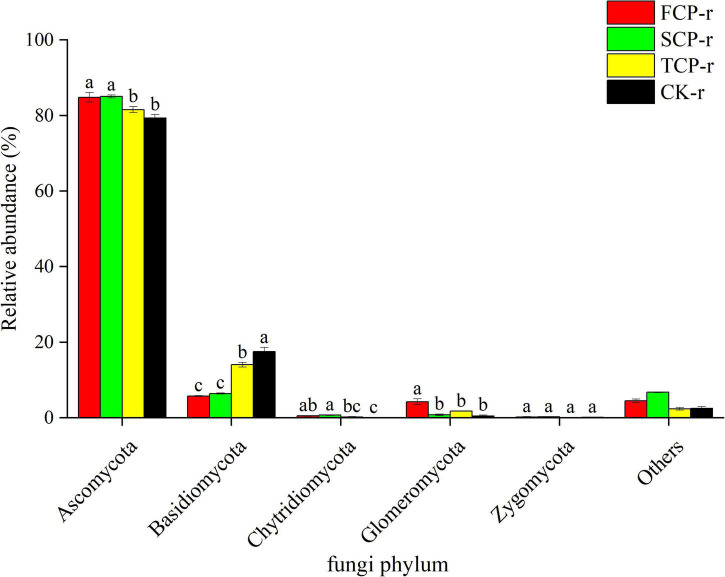
Relative abundance of predominant fungal community in successive generations within rhizosphere soil at the phyla level. Other stands for undefined categories. Value show means ± standard error (*n* = 3). Different lowercase letters show significant differences. CK, Nanmu seedings; FCP, first-generation seedings; SCP, second-generation seedings; TCP, third-generation seedings. r, rhizosphere soil.

Among all treatments at soil types, the fungal communities with the highest relative abundance at the phylum level were Ascomycota (57.63–97.38%) and Basidiomycota (5.74–32.89%) (Figure 3 and Supplementary Figure 2). The different successive generations had different effects on bacteria and fungi at the phylum level. Particularly, in all soil types, the relative abundance of Ascomycota was noticeably lower in TCP than in SCP (Figure 3 and Supplementary Figure 2), but its relative abundances in FCP and SCP were not substantially different (Figure 3). Compared with FCP and TCP, the relative abundance of Basidiomycota was markedly lesser in SCP but was not significant difference between FCP and TCP in bulk soils (Supplementary Figure 2). In addition, Glomeromycota and Zygomycota differed remarkably in FCP and SCP at the phylum level, whereas Chytridiomycota had no visible differences among all treatments (Supplementary Figure 2). In the rhizosphere soil, the relative abundance of Basidiomycota was obviously higher in TCP and CK than in FCP and SCP, but no significant difference was found between FCP with SCP (Figure 3). Moreover, the relative abundances of Chytridiomycota and Zygomycota did not remarkably change with the replant (Figure 3).

**FIGURE 4:**
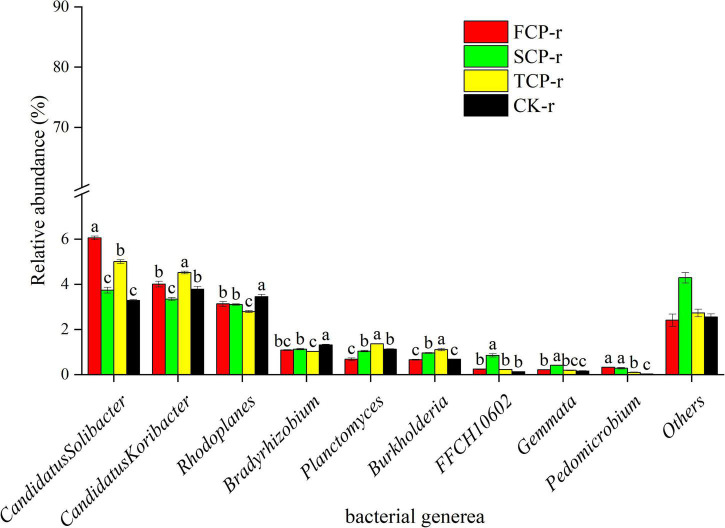
Relative abundance of predominant bacterial community in successive generations within rhizosphere soil at the genus level. Other stands for undefined categories. Value show means ± standard error (*n* = 3). Different lowercase letters show significant differences. CK, Nanmu seedings; FCP, first-generation seedings; SCP, second-generation seedings; TCP, third-generation seedings. r, rhizosphere soil.

**FIGURE 5:**
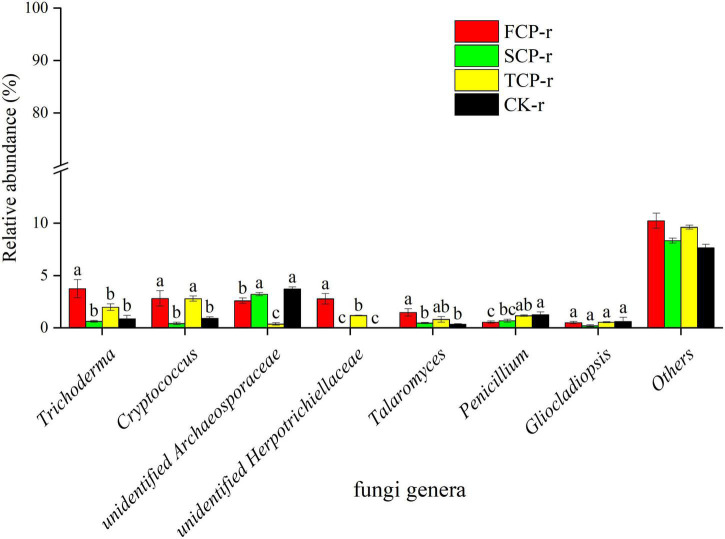
Relative abundance of predominant fungal community in successive generations within rhizosphere soil at the genus level. Other stands for undefined categories. Value show means ± standard error (*n* = 3). Different lowercase letters show significant differences. CK, Nanmu seedings; FCP, first-generation seedings; SCP, second-generation seedings; TCP, third-generation seedings. r, rhizosphere soil.

The bacteria with the highest relative abundances at the genus level were *Candidatus Solibacter*, *Candidatus Koribacter*, and *Rhodoplanes* (Figure 4 and Supplementary Figure 3), whereas the highest relative abundance of fungi at the genus level was found in *Trichoderma* and *Cryptococcus* (Figure 5 and Supplementary Figure 4).

**FIGURE 6:**
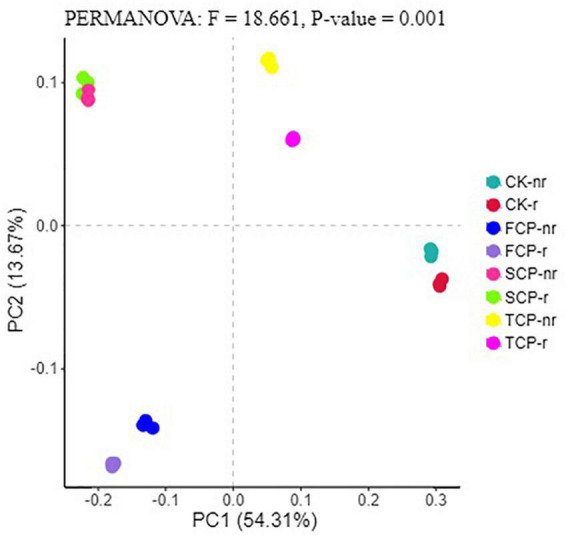
Principal coordinate analysis (PCoA) for bacterial in different fir seedings and soil types (using combined rhizosphere soil and bulk soil data). Data were obtained from different successive planting generations and soil types CK, Nanmu seedings; FCP, first generation seedings; SCP, second-generation seedings; TCP, third-generation seedings; r, rhizosphere soil; nr, bulk soil.

**FIGURE 7:**
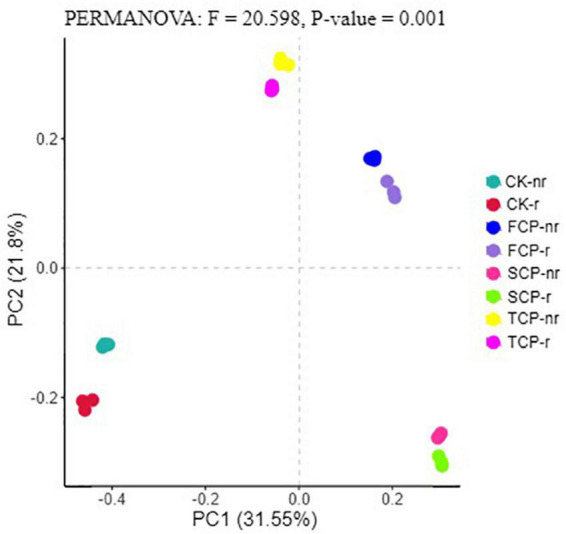
Principal coordinate analysis (PCoA) for fungal in different fir seedings and soil types (using combined rhizosphere soil and bulk soil data). Data were obtained from different successive planting generations and soil types CK, Nanmu seedings; FCP, first generation seedings; SCP, second-generation seedings; TCP, third-generation seedings; r, rhizosphere soil; nr, bulk soil.

Following PCoA analyses, the ordination diagram for the soil samples under the CK treatment and successive planting of fir showed independent clusters, and PERMANOVA analysis revealed significant differences in the microbial community structure between treatments (Figures 6, 7 and Supplementary Table 7).

### Correlation between soil microbial diversity and chemical properties

**FIGURE 8:**
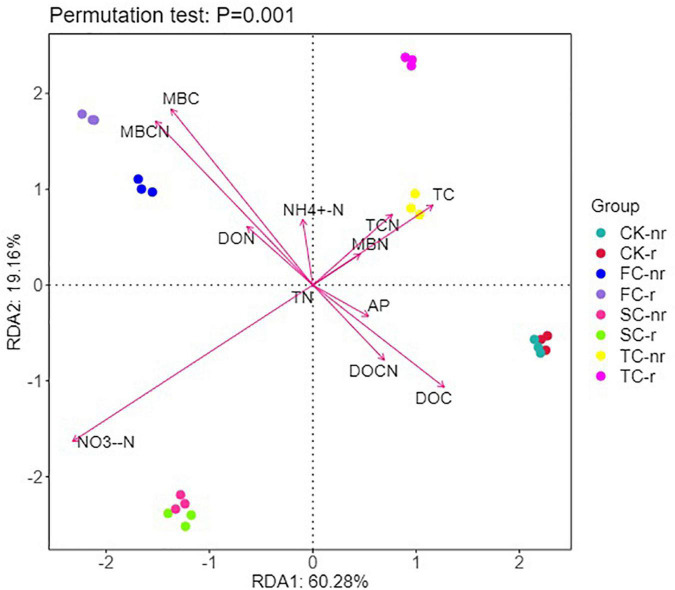
Redundancy analysis (RDA) explains the impact of environmental variables (arrows) on the structure of bacterial community in different seedings and soil types (using rhizosphere soil and bulk soil data). The values for axis 1 and axis 2 are the percentages explained for the correspondent axes. Data were obtained from different successive planting generations and soil types (CK, Nanmu seedings; FCP, first-generation seedings; SCP, second-generation seedings; TCP, third-generation seedings; r, rhizosphere soil; nr, bulk soil).

Based on the RDA characteristic values, axes 1 and 2 explained 60.28 and 19.16% of the variation in the bacterial community of the two soil types, respectively (Figure 8). In addition, Monte Carlo permutation tests indicated the significant impacts of key soil abiotic properties on the structure of the bacterial and fungal community (Supplementary Table 6). RDA results elucidated that MBC, MBCN, NO_3_^–^-N, DOC, and TC were the key supporters of the shift in soil bacterial communities (Figure 8). MBC, MBCN, DOC, DOCN, and AP were remarkably negatively correlated with the diversity indices (Chao1, ACE, and Shannon indices), and TC, TCN, and NO_3_^–^-N were remarkably positively correlated with the diversity indices in rhizosphere soil (Supplementary Table 5). Moreover, TC, DON, and AP were substantially negatively correlated with the diversity indices (Chao1, ACE, and Shannon indices), and MBC, MBCN, NH_4_^+^-N, and NO_3_^–^-N were considerably positively correlated with the diversity indices in bulk soil (Supplementary Table 5).

**FIGURE 9:**
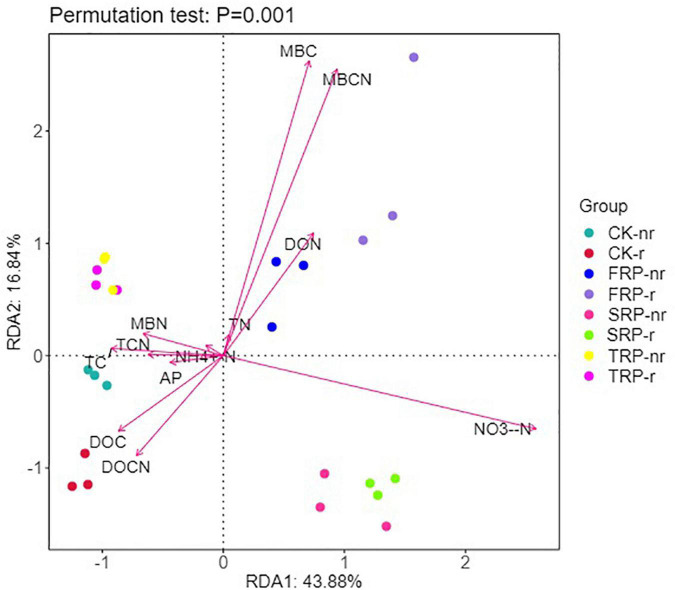
Redundancy analysis (RDA) explains the impact of environmental variables (arrows) on the structure of fungal community in different seedings and soil types (using rhizosphere soil and bulk soil data). The values for axis 1 and axis 2 are the percentages explained for the correspondent axes. Data were obtained from different successive planting generations and soil types (CK, Nanmu seedings; FCP, first-generation seedings; SCP, second-generation seedings; TCP, third-generation seedings; r, rhizosphere soil; nr, bulk soil).

Similarly, the RDA results showed that axes 1 and 2 explained 43.88 and 16.84% of the variation in the fungal communities of the two soil types, respectively (Figure 9). All of soil abiotic components, namely, MBC, MBCN, NO_3_^–^-N, DOC, DON, and DOCN occupied the largest proportion of the alteration in soil fungal communities (Figure 9). TC, TCN, DOCN, and NH_4_^+^-N were remarkably positively correlated with the diversity indices (Chao1, ACE, and Shannon indices), and MBC, MBCN, DON, NO_3_^–^-N, and AP were remarkably negatively correlated with the diversity indices in rhizosphere soil (Supplementary Table 5). DOCN, NH_4_^+^-N, TC, TN, and TCN were remarkably positively correlated with the diversity indices (Chao1, ACE, and Shannon indices), and MBC, MBCN, NO_3_^–^-N, and AP were notably negatively correlated with diversity indices in bulk soil (Supplementary Table 5).

## Discussion

### Effects of successive planting on soil microbial community diversity

Forest ecosystems differ because of stand structure, litter properties, and seasonal changes (Ren et al., 2017). These differences may affect soil bacterial and fungal communities. Soil microbial diversity including richness indices (Chao1, ACE) and diversity indices (Shannon, Simpson) (Du et al., 2017). The results of the current study indicate that the richness and diversity indices of bacteria and fungi in rhizosphere soils were lower in TCP than in FCP and SCP (Tables 2, 3). Our research results are partially in line with a previous study, which revealed that as *Cunninghamia lanceolata* replants, bacterial diversity declines, but the fungal diversity becomes richer within SCP and TCP compared with FCP in rhizosphere soil (Wu et al., 2017). By contrast, some researchers reported a decline in fungal diversity in the pure planted stands of *C. equisetifolia*, which is in line with our study (Zhou et al., 2019). The opposite outcomes for fungi may be attributed to the use of different research methods. Wu et al. (2017) used PLFAs to study changes in soil microorganisms, whereas Zhou et al. (2019) used the pyrophosphate sequencing technique. Although PLFA is a fast quantitative analysis method for soil microbial structure, new-generation sequencing technologies are more credible in exploring microbial communities (Liu et al., 2020), as they deliver information at a higher quality and resolution, and they not constrained by certain microbial taxa (Wang et al., 2022), such as PLFA. In addition, this study also found that bacterial (Shannon index) and fungi diversity (ACE index) was inferior in the rhizosphere soil than in the bulk soil (Tables 2, 3). This outcome may be influenced by plant root activity (Cao et al., 2017), which resulted in the critical distinction among the physicochemical properties of rhizosphere and bulk soils. For example, DOC and NO_3_^–^-N contents in rhizosphere soil were remarkably lower than those in bulk soil (Table 1), which further led to alterations in the composition of the rhizosphere soil’s microbial community (Sun et al., 2020). For a long time, biodiversity has been seen as a decisive driver of ecosystem function, whereas the biodiversity of the subsurface has long been neglected because of its high redundancy. Increasing evidence shows that the missing microbial diversity will damage a variety of ecosystem activities, such as litter breakdown (Veen et al., 2021) and nutrient uptake (Xu et al., 2021). Prior analysis has shown that soil biodiversity can promote litter decomposition, minimize nutrient leaching, and contribute to the maintenance of nutrient turnover in the surface and subsurface parts of the microbial community (Ritter et al., 2018). Most former studies believed that the decline in productivity in fir plantations is attributed to the decline in soil nutrients (Farooq et al., 2019), but the problem cannot simply be solved by fertilization (Wu et al., 2017). This finding also demonstrates the importance of microbes in resolving this problem. Our research results answered the previous hypothesis by showing that the richness and diversity of bacteria and fungi decreased in TCP compared with those in FCP and SCP (Tables 2, 3). Unexpectedly, for the most part, no remarkable rise or decline in soil TN and MBN contents was observed in FCP and SCP (Table 1). However, soil NO_3_^–^-N in SCP and TCP remarkably decreased compared with that in FCP at both sampling soils (Table 1). Therefore, soil nitrogen, especially mineral nitrogen, may be a more restricted nutrient than TC and AP in the subtropical regions of China, considering its unavailability in *C. lanceolata* plantations (Yu et al., 2021).

### The change of soil bacteria and fungi community composition

Deep sequencing results showed that Basidiomycota and Ascomycota were the principal groups among the fungal phyla, and Acidobacteria, Proteobacteria, Actinobacteria, and Planctomycetes were the dominant groups among the bacterial phyla (Figures 1, 2). These results were consistent with previous studies (Wang et al., 2018; Liu et al., 2020). Our studies suggest that the relative abundances of the dominant communities at the fungal phylum level changed considerably with successive planting (Figure 3 and Supplementary Figure 2). Related studies showed that Mucoromycota was the first immigrant among the fungal communities, followed by Ascomycota (Torres et al., 2005), which is regarded as a degrader of cellulose or sugar fungi, but has restricted ability to degrade lignin (Osono, 2007). However, Basidiomycota dominated the subsequent litter decomposition because of its ability to degrade recalcitrant lignin in the litter (Osono, 2020).

The litterfall of Chinese fir has its own morphological and chemical features that result in its slow decomposition and nutrient cycling processes (Wang et al., 2019). Fungi have an important role in promoting litter decomposition and carbon cycling in forest ecosystems due to their broader ability to take up carbon (Lodato et al., 2021). In this study, the minimum relative abundance of Ascomycota and the maximum relative abundance of Basidiomycota in the TCP (Figure 3) suggest that potential change in the carbon composition of fir plantation forests over time. Specially, stubborn carbon pooling occurred in the soil as the successive generations increased and caused an elevation in the relative abundance of lignin degraders (Basidiomycota) and a decrease in the relative abundance of sugar fungi (Ascomycota). The results suggested that the successive planting of Chinese fir plantations reduce the source of soil carbon, which was manifested by the remarkably lower soil TC content in TCP compared with those in FCP and SCP (Table 1). This finding is consistent with prior research, which found that the soil carbon metabolic activity in the successive rotations of fir plantations is in the order: FCP > SCP > TCP (Wu et al., 2017).

*Penicillium* was found in the two soil types and was remarkably higher in TCP than in FCP and SCP (Figure 5 and Supplementary Figure 4). Studies have shown that *Penicillium* can cause plant diseases. For example, the green and blue mold caused by *Penicillium digitatum* and *Penicillium italicum* are the two main diseases of citrus after picking that cause great economic losses (Chen et al., 2019). In addition, we also found that the relative abundances of *Trichoderma* in SCP and TCP were markedly lower than that in FCP (Figure 5). According to earlier reports, *Trichoderma* can act as an inhibitor of soil fungal diseases (Fiorentino et al., 2018; Zhang et al., 2018).

This study found that the relative abundance of Acidobacteria ranks at the top of the bacterial categories in rhizosphere and bulk soils (Figure 2 and Supplementary Figure 1). Earlier works have shown that Acidobacteria is the primary taxon in highly acidic soils (4 < pH < 5) (Lauber et al., 2009), which is consistent with our study. Successive planting had a remarkable impact on the relative abundance of primary bacterial phyla (Acidobacteria, Proteobacteria, and Actinobacteria), which is most likely related to soil pH. Previous studies by our group showed that soil pH decreased from 3.97 to 3.84 with the increases of successive generations, soil pH was markedly lower in TCP than in FCP and SCP, and the difference in soil pH between FCP and SCP was significant (Wei et al., 2016). The rest of the studies also showed that soil pH is a key factor affecting soil bacterial communities (Mayerhofer et al., 2021). This study found that the relative abundances of Actinobacteria and Proteobacteria in the rhizosphere soil of TCP was higher than those of FCP, and the same situation also occurred in the bulk soil (Figure 2 and Supplementary Figure 1). The former may be due to a large amount of litter in the TCP, because Actinobacteria can use lignin-derived compounds to participate in litter decomposition (Kirby, 2005), while the latter may be associated with a broader ecological niche, Proteobacteria are most common in terrestrial soils (Kim et al., 2021). *Burkholderia* was found in the two soil types and was remarkably higher in TCP than in FCP and SCP (Figure 4 and Supplementary Figure 3). Some studies have shown that *Burkholderia* can cause onion stem rot (Yuan et al., 2007; Tsuji and Kadota, 2020). Notably, inadequate or incorrect taxonomic annotation of species in international DNA databases due to current technical limitations has greatly limited our identification of bacteria and fungi.

### Environmental factors influencing soil microbial community structure

RDA, correlation analysis and Monte Carlo permutation tests showed that changes in the nutrient environment of successive seedings had different effects on the changes in soil bacterial and fungal communities (Figures 8, 9 and Supplementary Tables 5, 6). In general, soil NO_3_^–^-N, DOC, DOCN, MBC, and MBCN played an extremely important role in driving changes in soil microbial communities, but the bacterial and fungal communities differed in size and direction.

Soil DOC were important in shaping the microbial community composition (Cookson et al., 2007), and Pearson correlation analysis showed that DOC was significantly and negatively correlated with bacterial community alpha diversity in rhizosphere soils (Supplementary Table 5). We speculate that this may be caused by a significant increase in the relative abundance of *Actinobacteria*. Relevant studies point out that *Actinobacteria* tend to utilize recalcitrant carbon sources and are insensitive to DOC uptake (Verzeaux et al., 2016).

In additions, the DOC content of rhizosphere soils in SCP and TCP was significantly higher than that in FCP (Table 1), which may be related to the plant root activity (Philippot et al., 2013; Ren et al., 2018). Chinese fir in SCP and TCP are in the fast-growing and productive period at this time, and their root activity is higher than that in FCP, so they will produce a large amount of root exudates. According to related studies, plant root exudates contain low molecular mass compounds and sugar polymer, which can be used as carbon sources by rhizosphere microorganisms (Philippot et al., 2013). Thus, resulting in significantly higher DOC content of rhizosphere soils in SCP and TCP than in FCP.

Although soil MBC accounts for only 2% of SOC, MBC is important for soil carbon conversion and cycling. In the present study, the content of MBC in rhizosphere soil is higher than that in bulk soil (Table 1). Obviously, the greater diversity and richness of microbial and activity in rhizosphere soils is clearly the cause of this (Baldrian, 2017). The sequencing results showed that the bacterial and fungal richness index (Chao1 index) was 6.82 and 0.82% higher and the diversity index (Shannon index) was 0.66 and 5.03% higher in the rhizosphere soil than in the bulk soil. Moreover, we also found a significant negative correlation between MBC and bacterial and fungal communities, indicating that MBC is an important factor limiting microbial activity. This opinion is supported by Jing et al. (2020). They pointed out that MBC is an essential factor affecting microbial enzyme activity in temperate forest soils. Nitrogen is the most important nutrient element for life activities; NH_4_^+^–N and NO_3_^–^-N are the most convenient available nitrogen source for microorganisms (Huhe et al., 2017). Our study showed that NO_3_^–^-N was strongly positively correlated with the bacterial community, and NH_4_^+^-N was strongly positively correlated with the fungal community (Supplementary Table 5).

## Conclusion

Overall, the study provides a clear indication of the decline in bacterial and fungal diversity in TCP compared with those in FCP and SCP. Our findings suggest that successive planting remarkedly alters the relative abundance of major bacterial and fungal groups. Moreover, the presence of pathogenic microorganisms (*Penicillium* and *Burkholderia*) and the reduction of beneficial fungi (*Trichoderma*) combined with the scarcity of available nitrogen. The combination of these factors is a key factor to the decrease in the productivity of continuous *C. lanceolata* plantations. MBC, DOC, nitrate nitrogen (NO_3_^–^-N) and stoichiometric ratio (MBCN, DOCN) also remarkably shaped bacterial and fungal communities.

## Data availability statement

The datasets presented in this study can be found in online repositories. The names of the repository/repositories and accession number(s) can be found below: NCBI, PRJNA861377.

## Author contributions

JC, GC, LW, and SC participated in the conception and design of this research. JC, GC, ZJ, XZ, and FM participated in the site work. JC and ZD organized the database and wrote the first draft of the manuscript. JC, JS, and LW completed the data analysis. GC and SC took primary responsibility for communication with the journal and editorial office during publication. All authors contributed to the manuscript revision, read and approved the submitted version.

## Footnotes

1
https://mothur.org/wiki/download_mothur/

2
http://www.canoco5.com

## Supplementary material

Supplementary Figure 1 Relative abundance of the most abundant bacterial group in different treatments in bulk soil at the phylum level. Others represented unclassified groups. Data are means ± standard error (*n* = 3). Significant differences are indicated with distinct lowercase letters. CK, Nanmu seedings; FCP, first-generation seedings; SCP, second-generation seedings; TCP, third-generation seedings; nr, bulk soil.

Supplementary Figure 2 Relative abundance of the most abundant fungal group in different treatments in bulk soil at the phylum level. Others represented unclassified groups. Data are means ± standard error (*n* = 3). Significant differences are indicated with distinct lowercase letters. CK, Nanmu seedings; FCP, first-generation seedings; SCP, second-generation seedings; TCP, third-generation seedings; nr, bulk soil.

Supplementary Figure 3 Relative abundance of the most abundant bacterial group in different treatments in bulk soil at the genus level. Others represented unclassified groups. Data are means ± standard error (*n* = 3). Significant differences are indicated with distinct lowercase letters. CK, Nanmu seedings; FCP, first-generation seedings; SCP, second-generation seedings; TCP, third-generation seedings; nr, bulk soil.

Supplementary Figure 4 Relative abundance of the most abundant fungal group in different treatments in bulk soil at the genus level. Others represented unclassified groups. Data are means ± standard error (*n* = 3). Significant differences are indicated with distinct lowercase letters. CK, Nanmu seedings; FCP, first-generation seedings; SCP, second-generation seedings; TCP, third-generation seedings; nr, bulk soil.
