# Supplementary material for: Variations of rhizosphere and bulk soil microbial community in successive planting of Chinese fir (Cunninghamia lanceolata)
Source: Front Plant Sci. 2022 Aug 12;13:954777. doi: 10.3389/fpls.2022.954777 (PMC9411970; doi:10.3389/fpls.2022.954777)
Supplement: Supplementary file 1 [file Data_Sheet_1.zip › Supplementary Tables/Table S1.docx]

**Table S1** Characteristics of sampling site

| Characteristics | FCP | SCP | TCP | CK |
| --- | --- | --- | --- | --- |
| Plantation age/a | 102 | 24 | 25 | 46 |
| Aspect | North-west | North-west | North-west | North-west |
| Slope/ (°) | 20 | 26 | 26 | 15 |
| Average height/m | 32.7 | 12.9 | 12.4 | 17.5 |
| Average DBH/cm | 33.5 | 12.1 | 11.5 | 26.4 |
| Stand density/ (stem hm^-2^) | 1009 | 2640 | 2775 | 2250 |
| Canopy density | 0.62 | 0.82 | 0.81 | 0.84 |
| Understory Vegetation | *Maesa japonica* | *Maesa japonica* | *Maesa japonica* | *Maesa japonica* |
|  | *Ficus hirta* | *Rubus buergeri* | *Ficus hirta* | *Lophatherum gracile* |
|  | *Rubus buergeri* | *Parathelypteris glanduligera* | *Dicranopteris dichotoma* | *Woodwardia japonica* |
|  | *Dicranopteris dichotoma* | *Athyrium iseanum* | *Woodwardia japonica* |  |
|  | *Polypodiodes niponica* | *Woodwardia japonica* |  |  |
|  | *Woodwardia japonica* |  |  |  |
