# Supplementary material for: Variations of rhizosphere and bulk soil microbial community in successive planting of Chinese fir (Cunninghamia lanceolata)
Source: Front Plant Sci. 2022 Aug 12;13:954777. doi: 10.3389/fpls.2022.954777 (PMC9411970; doi:10.3389/fpls.2022.954777)
Supplement: Supplementary file 1 [file Data_Sheet_1.zip › Supplementary Tables/Table S2.docx]

**Table S2** *P* values from two-way ANOVA (with repeated measure) of effects of successive planting generations (R), soil type (T) and their interaction (R×T) on soil physiochemical properties.

| Source | TC | TN | TCN | DOC | DON | DOCN | MBC | MBN | MBCN | NH_4_^+^-N | NO_3_^-^-N | AP |
| --- | --- | --- | --- | --- | --- | --- | --- | --- | --- | --- | --- | --- |
| R | <0.001 | 0.823 | 0.002 | <0.001 | 0.58 | 0.002 | <0.001 | 0.419 | <0.001 | <0.001 | <0.001 | <0.001 |
|  | *** |  | ** | *** |  | ** | *** |  | *** | *** | *** | *** |
| T | 0.799 | 0.71 | 0.523 | <0.001 | 0.28 | 0.004 | <0.001 | 0.427 | <0.001 | <0.001 | 0.13 | <0.001 |
|  |  |  |  | *** |  | ** | *** |  | *** | *** |  | *** |
| R×T | 0.31 | 0.264 | 0.402 | 0.835 | 0.379 | 0.101 | 0.006 | 0.918 | 0.59 | 0.199 | 0.001 | 0.061 |
|  |  |  |  |  |  |  | ** |  |  |  | ** |  |

Abbreviations: TC, total carbon; TN, total nitrogen; TCN, TC/TN ratio; DOC, dissolved organic carbon; DON, dissolved organic nitrogen; DOCN, DOC/DON ratio; MBC, microbial biomass carbon; MBN, microbial biomass nitrogen; MBCN, MBC/MBN ratio; NH_4_^+^-N, ammonium nitrogen; NO_3_^-^-N, nitrate nitrogen; AP, available phosphorus. Significance levels: **P* < 0.05, ***P* < 0.01, ****P* < 0.001.
