# Supplementary material for: Variations of rhizosphere and bulk soil microbial community in successive planting of Chinese fir (Cunninghamia lanceolata)
Source: Front Plant Sci. 2022 Aug 12;13:954777. doi: 10.3389/fpls.2022.954777 (PMC9411970; doi:10.3389/fpls.2022.954777)
Supplement: Supplementary file 1 [file Data_Sheet_1.zip › Supplementary Tables/Table S3.docx]

**Table S3** Total raw and final sequence numbers for each seedings.

| Soil type | Treatment | Sample | Total Raw16S *r*RNA sequences | Clean 16S *r*RNA sequences | OTUs  3%cut-off | Total Raw ITS sequences | Clean ITS sequences | OTUs  3%cut-off |
| --- | --- | --- | --- | --- | --- | --- | --- | --- |
| Rhizosphere soil | FCP | DecFCP1 | 76099 | 58691 | 7440 | 91488 | 15650 | 1012 |
|  |  | DecFCP2 | 91942 | 70565 | 9536 | 84799 | 15136 | 1058 |
|  |  | DecFCP3 | 86524 | 67338 | 9631 | 83978 | 13340 | 988 |
|  | SCP | DecSCP1 | 87235 | 69768 | 8308 | 80913 | 16168 | 1395 |
|  |  | DecSCP2 | 81208 | 65095 | 7787 | 85726 | 17449 | 1521 |
|  |  | DecSCP3 | 87811 | 70018 | 8003 | 85691 | 17321 | 1533 |
|  | TCP | DecTCP1 | 83758 | 64957 | 7068 | 82497 | 14957 | 1292 |
|  |  | DecTCP2 | 91213 | 70189 | 7705 | 81137 | 15053 | 1244 |
|  |  | DecTCP3 | 86843 | 67352 | 7729 | 88510 | 16411 | 1255 |
|  | CK | DecCK1 | 80973 | 59891 | 5408 | 91615 | 16118 | 1084 |
|  |  | DecCK2 | 86409 | 64326 | 5847 | 80889 | 12976 | 970 |
|  |  | DecCK3 | 86020 | 64076 | 5876 | 81846 | 14830 | 1049 |
| Bulk soil | FCP | DecFCP1 | 79565 | 62419 | 7747 | 83926 | 14578 | 1090 |
|  |  | DecFCP2 | 85729 | 67269 | 8182 | 88620 | 15632 | 1089 |
|  |  | DecFCP3 | 91949 | 71937 | 8543 | 87493 | 15594 | 1037 |
|  | SCP | DecSCP1 | 85074 | 67436 | 8035 | 84133 | 14895 | 1349 |
|  |  | DecSCP2 | 83486 | 66133 | 7943 | 88842 | 16111 | 1380 |
|  |  | DecSCP3 | 84109 | 66841 | 8038 | 82339 | 15141 | 1356 |
|  | TCP | DecTCP1 | 85109 | 66938 | 8855 | 85397 | 15074 | 1314 |
|  |  | DecTCP2 | 80144 | 63861 | 8597 | 90664 | 16921 | 1334 |
|  |  | DecTCP3 | 80254 | 63929 | 8647 | 81619 | 14609 | 1263 |
|  | CK | DecCK1 | 83578 | 62451 | 6798 | 86859 | 16680 | 1309 |
|  |  | DecCK2 | 85109 | 63591 | 6759 | 83259 | 15504 | 1299 |
|  |  | DecCK3 | 83665 | 63036 | 6614 | 86331 | 16691 | 1350 |

^a^ Soil type for each treatment, Dec means detected sample, CK, Nanmu seedings; FCP, first-generation seedings; SCP, second-generation seedings; TCP, third-generation seedings.
