# Supplementary material for: Variations of rhizosphere and bulk soil microbial community in successive planting of Chinese fir (Cunninghamia lanceolata)
Source: Front Plant Sci. 2022 Aug 12;13:954777. doi: 10.3389/fpls.2022.954777 (PMC9411970; doi:10.3389/fpls.2022.954777)
Supplement: Supplementary file 1 [file Data_Sheet_1.zip › Supplementary Tables/Table S4.docx]

**Table S4** *P* values from two-way ANOVA (with repeated measure) of effects of successive planting generations (R), soil type (T) and their interaction (R×T) on soil fungal and bacterial diversity indices.

| Source | Fungi | | | | Bacteria | | | |
| --- | --- | --- | --- | --- | --- | --- | --- | --- |
|  | OTUs | Chao1 | ACE | Shannon | OTUs | Chao1 | ACE | Shannon |
| R | <0.001*** | 0.002* | <0.001*** | <0.001*** | 0.305 | 0.032* | 0.007* | <0.001*** |
| T | 0.633 | 0.881 | 0.349 | <0.001*** | 0.576 | 0.389 | 0.239 | <0.001*** |
| R×T | 0.006** | 0.205 | 0.039* | <0.001*** | 0.37 | 0.011* | 0.006* | <0.001*** |

OTUs, operational taxonomic units (97% similarity). Significance levels: **P* < 0.05, ***P* < 0.01, ****P* < 0.001.
