# Supplementary material for: Variations of rhizosphere and bulk soil microbial community in successive planting of Chinese fir (Cunninghamia lanceolata)
Source: Front Plant Sci. 2022 Aug 12;13:954777. doi: 10.3389/fpls.2022.954777 (PMC9411970; doi:10.3389/fpls.2022.954777)
Supplement: Supplementary file 1 [file Data_Sheet_1.zip › Supplementary Tables/Table S5.docx]

**Table S5** Pearson correlations between soil abiotic properties and alpha diversity indices of bacterial and fungal communities in rhizosphere soil and bulk soil.

|  | indices | soil type | TC | TN | TCN | MBC | MBN | MBCN |
| --- | --- | --- | --- | --- | --- | --- | --- | --- |
| Bacterial community | OTUs | rhizosphere soil | 0.331 | -0.389 | 0.426 | -0.393 | -0.28 | -0.293 |
|  | Chao1 |  | 0.155 | -0.262 | 0.24 | -0.127 | -0.304 | -0.049 |
|  | ACE |  | 0.107 | -0.217 | 0.185 | -0.028 | -0.286 | 0.036 |
|  | Shannon index |  | 0.621* | -0.452 | 0.668* | -0.722** | -0.051 | -0.640* |
|  | OTUs | bulk soil | -0.029 | -0.234 | 0.018 | -0.110 | -0.380 | 0.018 |
|  | Chao1 |  | -0.435 | -0.173 | -0.395 | 0.433 | -0.181 | 0.490 |
|  | ACE |  | -0.624* | -0.210 | -0.575 | 0.637* | -0.088 | 0.655* |
|  | Shannon index |  | 0.378 | -0.051 | 0.379 | -0.559 | -0.365 | -0.427 |
| Fungal community | OTUs | rhizosphere soil | 0.595* | 0.415 | 0.489 | -0.628* | -0.018 | -0.617* |
|  | Chao1 |  | 0.414 | 0.379 | 0.319 | -0.444 | -0.033 | -0.450 |
|  | ACE |  | 0.449 | 0.445 | 0.336 | -0.562 | 0.069 | -0.593* |
|  | Shannon index |  | 0.771** | 0.315 | 0.690* | -0.909** | 0.015 | -0.901** |
|  | OTUs | Bulk soil | 0.330 | 0.707* | 0.162 | -0.224 | 0.325 | -0.311 |
|  | Chao1 |  | 0.574 | 0.724** | 0.395 | -0.563 | 0.015 | -0.538 |
|  | ACE |  | 0.717** | 0.431 | 0.605* | -0.827** | -0.100 | -0.776** |
|  | Shannon index |  | 0.458 | 0.531 | 0.333 | -0.379 | 0.455 | -0.509 |

to be continued

|  | indices | soil type | DOC | DON | DOCN | NH_4_^+^-N | NO_3_^-^-N | AP |
| --- | --- | --- | --- | --- | --- | --- | --- | --- |
| Bacterial community | OTUs | rhizosphere soil | -0.865** | 0.298 | -0.738** | -0.016 | 0.567 | -0.766** |
|  | Chao1 |  | -0.713** | 0.290 | -0.619** | -0.305 | 0.760** | -0.625* |
|  | ACE |  | -0.643* | 0.300 | -0.575 | -0.405 | 0.801** | -0.554 |
|  | Shannon index |  | -0.819** | 0.322 | -0.753** | 0.453 | 0.028 | -0.808* |
|  | OTUs | bulk soil | -0.225 | -0.474 | 0.304 | 0.585* | 0.469 | -0.449 |
|  | Chao1 |  | 0.211 | -0.355 | 0.328 | 0.454 | 0.717** | 0.122 |
|  | ACE |  | 0.310 | -0.146 | 0.187 | 0.287 | 0.824** | 0.315 |
|  | Shannon index |  | -0.390 | -0.578* | 0.368 | 0.680* | -0.002 | -0.758** |
| Fungal community | OTUs | rhizosphere soil | 0.141 | -0.752** | 0.682* | 0.898* | -0.401 | -0.371 |
|  | Chao1 |  | 0.224 | -0.676* | 0.600* | 0.847** | -0.142 | -0.209 |
|  | ACE |  | 0.153 | -0.635* | 0.555 | 0.844** | -0.272 | -0.281 |
|  | Shannon index |  | -0.327 | -0.357 | 0.225 | 0.291 | -0.855** | -0.626* |
|  | OTUs | bulk soil | 0.551 | -0.443 | 0.578* | 0.602* | -0.396 | 0.329 |
|  | Chao1 |  | 0.004 | -0.394 | 0.409 | 0.527 | -0.595* | -0.263 |
|  | ACE |  | -0.352 | -0.406 | 0.278 | 0.393 | -0.709** | -0.661** |
|  | Shannon index |  | 0.347 | -0.229 | 0.323 | 0.143 | -0.693* | 0.259 |

OTUs, operational taxonomic units (97% similarity). Abbreviation for soil chemical properties refers to Table 1. Significance levels: **P* < 0.05, ***P* < 0.01.
