# Supplementary material for: Variations of rhizosphere and bulk soil microbial community in successive planting of Chinese fir (Cunninghamia lanceolata)
Source: Front Plant Sci. 2022 Aug 12;13:954777. doi: 10.3389/fpls.2022.954777 (PMC9411970; doi:10.3389/fpls.2022.954777)
Supplement: Supplementary file 1 [file Data_Sheet_1.zip › Supplementary Tables/Table S6.docx]

**Table S6.** Monte Carlo permutation tests of soil environmental factors (abiotic properties) and bacterial and fungal structures. Significant correlations at the *p* < 0.05 level are indicated in bold. Abbreviations have the same meanings as in Table 1.

|  | *p value* | |
| --- | --- | --- |
|  | Bacteria | Fungi |
| TC | **0.017** | 0.055 |
| TN | 0.976 | 0.610 |
| TCN | 0.063 | 0.151 |
| MBC | **0.001** | **0.001** |
| MBN | 0.242 | 0.125 |
| MBCN | **0.001** | **0.001** |
| DOC | **0.008** | **0.033** |
| DON | 0.094 | **0.013** |
| DOCN | 0.068 | **0.028** |
| NO_3_^—^ | **0.001** | **0.001** |
| NH_4_^+^ | 0.165 | 0.611 |
| AP | 0.208 | 0.287 |
