# Supplementary material for: Variations of rhizosphere and bulk soil microbial community in successive planting of Chinese fir (Cunninghamia lanceolata)
Source: Front Plant Sci. 2022 Aug 12;13:954777. doi: 10.3389/fpls.2022.954777 (PMC9411970; doi:10.3389/fpls.2022.954777)
Supplement: Supplementary file 1 [file Data_Sheet_1.zip › Supplementary Tables/Table S7.docx]

**Table S7.** Pairwise comparison of soil bacterial and fungal community structure between each treatment in the PERMANOVA analysis (*p* values in parentheses; ** indicates *p*<0.01). CK: control.

|  | First | Second | Third |
| --- | --- | --- | --- |
| CK | 0.002^**^ | 0.002^**^ | 0.002^**^ |
| First |  | 0.002^**^ | 0.002^**^ |
| Second |  |  | 0.002^**^ |
| Third |  |  |  |
